# Temporal trends in age- and stage-specific incidence of colorectal adenocarcinomas in Germany

**DOI:** 10.1186/s12885-023-11660-1

**Published:** 2023-12-01

**Authors:** Annika Waldmann, Pia Borchers, Alexander Katalinic

**Affiliations:** Institute for Social Medicine and Epidemiology, University of Luebeck, Ratzeburger Allee 160, 23562 Luebeck, Germany

**Keywords:** Incidence, Epidemiology, Colorectal cancer, Time trends, Population-based data, cancer registry, Young adults

## Abstract

**Background:**

A national colorectal cancer (CRC) screening programme was launched in 2002 in Germany. A comprehensive evaluation of the programme effectiveness using real-world data is still lacking. In addition, there are regional reports on increasing colorectal cancer incidence in younger populations. Therefore, we aimed to describe and compare the overall, age- and stage-specific incidence trends for colorectal, colon and rectal cancer.

**Methods:**

We used data from seven population-based cancer registries in Germany. We report absolute and relative changes in incidence rates between the early screening phase (2003–2005) and the most recent time period available (2015–2017), as well as annual percent changes. We analysed incidences according to tumour site (colorectum, colon, and rectum) and to six age groups (young adults: 15–34, 35–39, 40–49, screening-entitled/older adults: 50–54, 55–69 and 70 + years old).

**Results:**

In our sample of 271,011 colorectal adenocarcinomas, about two-thirds were located in the colon and 95% of them occurred in the age group 50+ (50–54: 5%, 55–69: 32.8%, 70+: 57.2%). For the time period 2003–2005 the age-specific incidence rates of individuals in the age group 55–69 were about 76/100,00 for colon and 54/100,000 for rectal cancer (age group 70 + colon: 179/100,000; rectum: 84/100,000). The incidence rates in young adults were less than 13% of that of individuals in the age group 55–69 (< 5% of individuals aged 70+; <33% of individuals aged 50–54).

Over time, incidence decreased in individuals at the age of 55+, for all subsites considered as well as for early and late stage cancers (with few exceptions), while incidence of young adult CRC (both early and late stage) increased steepest in the youngest age groups. For late stage rectal cancer, a shift was observed in all age groups from UICC stage IV to stage III being the most frequent stage.

**Conclusions:**

Six years after the introduction of the national colonoscopy screening program, late stage CRC incidence began to decline substantially in the screening-eligible age groups (55-69, 70+). It is likely that this decline and the increase in early stage CRC observed in younger age groups can be attributed to the program. Long lasting public awareness campaigns for CRC screening might have led to opportunistic screening in younger adults. Whether these benefits outweigh the possible harm of screening in younger age groups remains unclear.

**Supplementary Information:**

The online version contains supplementary material available at 10.1186/s12885-023-11660-1.

## Background

Worldwide, colorectal cancer (CRC) contributes significantly to the overall cancer burden [1]. In Germany, CRC incidence ranks third in males after prostate and lung cancer and second in females after breast cancer. It is the third most frequent cause of death among the cancer related deaths [2, 3]. Incidence rates in 2019 were about one third higher in males than in women with age-adjusted rates of 49.8 and 31.9 per 100,000 (European Standard 1976). Recently, incidence trends for CRC in North America, Europe, China and Australia have been characterized by a decline in the overall population and in particular in individuals at the age of 50 or older i.e. in those who are eligible for CRC screening [1, 4–10]. Opposite to this, CRC incidence in younger adults has been increasing lately [4, 6, 7, 9–19].

The majority of CRC evolves from benign polyps. Progression to malignant lesions often spans over a prolonged period of time, typically at least a decade [20]. Screening for CRC is the only early detection method that can stop the progression of CRC, as there are procedures for detecting and removing pre-cancerous polyps or early stage malignant lesions [21]. Only recently, a pragmatic randomized controlled trial with a median follow-up time of 10 years showed that the risk of developing CRC was lower in participants who were invited for screening colonoscopy than in those who were not invited to screening [22].

The US Preventive Services Task Force recommends to offer CRC screening starting from the age of 45 until the age of 75 years [23]. In Germany, individuals starting from the age of 55 years on had been entitled since 2002 to quality-assured screening colonoscopies, performed twice with a ten-year interval between them. Additionally, individuals aged 50 to 54 years had the annual right to undergo examination of their stool with a guaiac faecal occult blood test (gFOBT). Upon reaching 55 years of age, this right changed to gFOBT conducted every other year, as an alternative to the colonoscopy. In the year 2018, the gFOBT was replaced by immunochemical faecal occult blood test (iFOBT) and the start age of offering CRC screening colonoscopies for men was lowered down to 50 years [24].

The primary preventive potential of CRC screening has been demonstrated through both population-based real-world and trial data including a pragmatic randomised controlled trial, as well as modelling studies [5, 7, 22, 25–31]. Although the evaluation of CRC screening appears positive at first glance, further research is needed. There is no comprehensive comparison between older individuals (aged 50–54, 55–69 and 70 + years) who are eligible for screening and younger individuals who are not eligible for screening - based on a large real-world database that considers the stage distribution and the location of the tumours. Additionally, current stage-specific incidence rates for CRC are lacking. Consequently, our objective is to examine how the patterns of CRC by age and stage have changed over time since the introduction of CRC screening in Germany.

## Methods

### Data source

We used a population-based data set provided by the Centre for Cancer Registry Data (ZfKD) at the Robert Koch Institute, Berlin, for our analysis. This data set is available upon request [32]. We included females and males with malignant neoplasms in the colon (ICD-10 C18), rectosigmoid (C19) or rectum (C20) from the diagnosis years 2003–2017 and with residence in the federal states of Bavaria, Bremen, Hamburg, Lower Saxony, Saarland, Schleswig-Holstein, and in the administrative district of Muenster in North Rhine-Westphalia (Supplemental Fig. 1). These regions include about 36% of the German population and offer a consistently high level of completeness over the mentioned time period.

Due to recent findings indicating that the incidence of carcinoids is rising more rapidly than that of adenocarcinomas among young individuals [17] and that appendiceal cancers could impact colon cancer incidence [9], we restricted our analysis to adenocarcinomas and tumour sites other than appendix (Supplemental File 1). We further excluded cases notified with stage 0 disease, and those occurring in persons 0–14 years at diagnosis. In addition, we excluded cases that have been notified to the registries only via death certificate (DCO cases) and that have not been confirmed histologically.

### Statistics

**Fig. 1:**
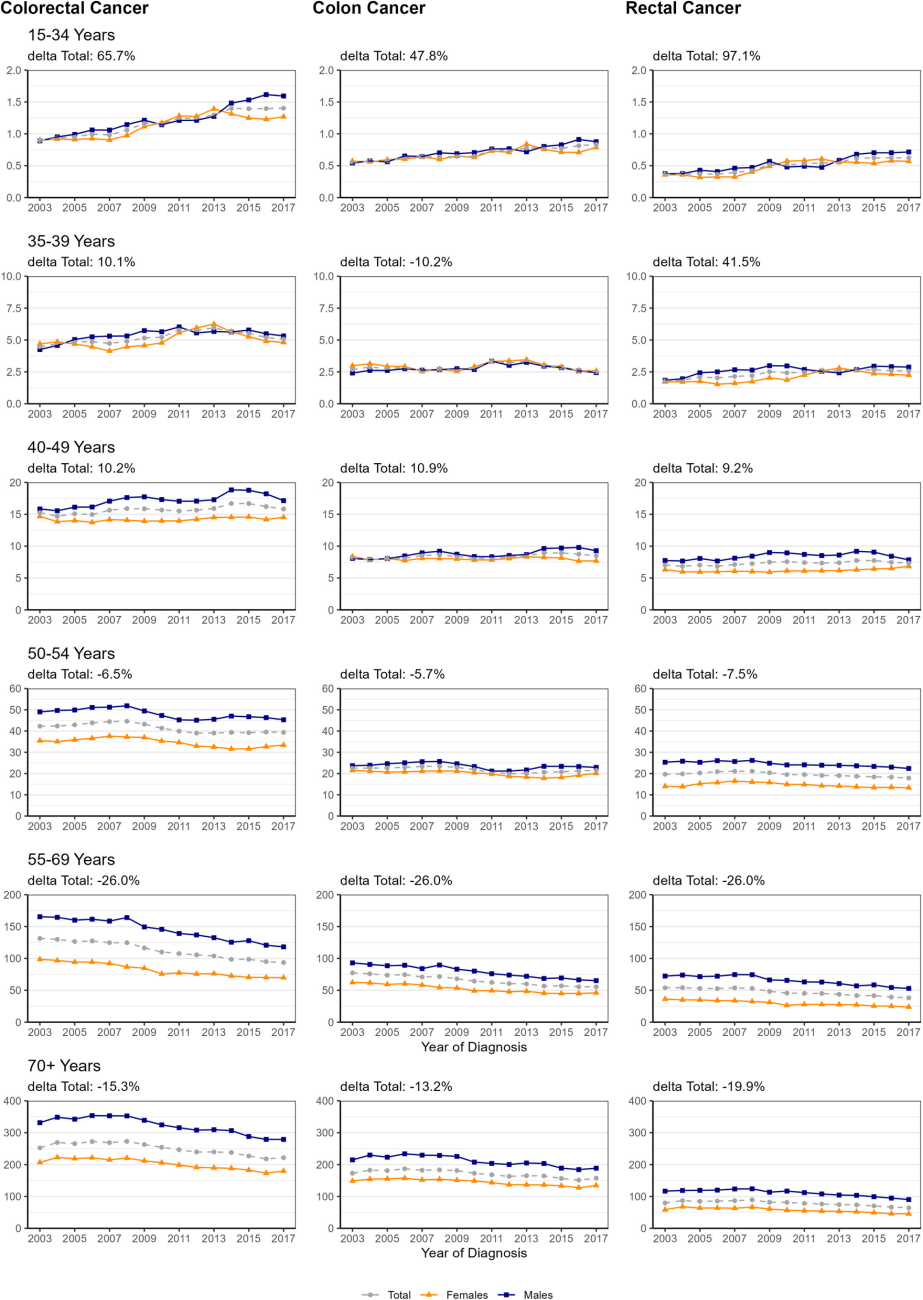
Time Trends of Age-Specific Incidence Rates According to Site, Age at Diagnosis & Sex. Age-specific incidence rates = Cases per 100,000; Rates for the age-groups 15-34 to 50-54 years = moving averages; delta Total = relative difference in age-specific incidence rates between the diagnosis period 2003-2005 and 2015-2017 as laid out in Table 2; Blue Squares = Males, Grey Circles = Both Sexes, Yellow Triangles = Females

We describe our sample using frequency distributions and common measures of descriptive statistics. Incidence is presented as age-specific as well as age-standardized incidence rates per 100,000 (European Standard 1976 and Segi’s World Standard) [33]. Results are given for the total population and differentiated by sex (female, male) and by age of diagnosis (six age groups: 15–34, 35–39, 40–49, 50–54, 55–69 and 70+). In addition, results are presented according to tumour site (colon and rectum and summarized as colorectum), time of diagnosis, and Union for International Cancer Control (UICC) stage (early stage: I & II vs. late stage: III & IV). As the number of incident cases was low for the age groups 15–34 to 40–49 years (Supplemental Table 1), we computed moving averages (3 years) for the incidence rates shown in Fig. 1.

Time trends are described as absolute and relative differences in rates for the early screening phase (mean of 2003–2005) compared to the most recent three years (mean of 2015–2017). We further conducted time trend analysis using Joinpoint regression models by applying an algorithm to define significant changes in time trends on a logarithmic scale. The annual percent change (APC) in each Joinpoint segment represents the percentage change in cancer incidence per year in the segment (SEER Joinpoint Software 4.9.1.0 [34]; model specifications are described in Supplemental File 2).

The proportion of missing information on the UICC stage was around 38%. Missing stages were imputed using multiple imputation (with chained equations, 5 imputations with 25 iterations, method polytomous regression; R package mice version 3.13.0 [35]). We used the open-source software R 4.1.0 for our analyses [36].

### Approval of study protocol and ethics approval

The study protocol, methodological and ethical aspects were discussed by and the protocol was approved by the scientific committee of the Centre for Cancer Registry Data (ZfKD) at the time of requesting the data. The permission to use the (anonymized) nationwide data set was granted on 25th June 2021 under the file number “5.03.04/0002#0083 − 0003”.

Research with anonymous data without patient contact was not subject to an ethics vote in Germany at the time of study protocol generation in 2021. However, ethical approval was subsequently obtained from the ethics committee of the University of Luebeck (31st Mai 2023 under the file number “2023 − 467”).

## Results

### Description of the study sample

For the time period 2003 to 2017, a total of 347,575 incident CRC cases were notified to the seven registries included in our study (cases over time by cancer registries are displayed in Supplemental Fig. 2). After excluding the DCO-cases (8.7%), cases without histological verification (11.3%), cases with histology other than adenocarcinoma (19.8%) or tumour location in the appendix (1.3%), a total of 271,011 cases remained in the sample for further analysis (Table 1).

**Table 1:** Description of colorectal cancer cases with diagnosis between 2003 and 2017 according to cancer registry

	Bavaria	Bremen	Hamburg	Lower Saxony	Muenster in North Rhine-Westphalia	Saarland	Schleswig-Holstein
**Total Number of Cases**	**140,249**	**8,032**	**17,974**	**99,448**	**32,644**	**13,954**	**35,274**
DCO-cases	14,176 (10.1)	322 (4.0)	1,458 (8.1)	8,682 (8.7)	1,755 (5.4)	494 (3.5)	3,410 (9.7)
Not histologically confirmed	15,697 (11.2)	557 (6.9)	2,646 (14.7)	13,002 (13.1)	2,509 (7.7)	844 (6.0)	3,852 (10.9)
Other than adenocarcinoma	27,451 (19.6)	1,439 (17.9)	4,595 (25.6)	17,915 (18.0)	7,682 (23.5)	3,147 (22.6)	6,481 (18.4)
Site: Appendix (C18.1)	1,946 (1.4)	112 (1.4)	224 (1.2)	1097 (1.1)	406 (1.2)	135 (1.0)	504 (1.4)
**Number of Cases Included for Analyses**	**110,722**	**6,410**	**12,963**	**77,927**	**24,402**	**10,582**	**28,005**
**Sex**							
Female	46,050 (41.6)	3,045 (47.5)	6,345 (48.9)	35,431 (45.5)	11,101 (45.5)	4,533 (42.8)	13,023 (46.5)
Male	64,672 (58.4)	3,365 (52.5)	6,618 (51.1)	42,496 (54.5)	13,301 (54.5)	6,049 (57.2)	14,982 (53.5)
**Age at Diagnosis**							
Median age (in years; 25^th^-75^th^ percentile)	71.6 (63-79)	73.2 (65-80)	72.4 (64-80)	72.5 (64-80)	73.1 (64-80)	71.9 (64-79)	72.2 (64-79)
15-34	521 (0.5)	22 (0.3)	91 (0.7)	340 (0.4)	76 (0.3)	26 (0.2)	120 (0.4)
35-39	670 (0.6)	36 (0.6)	87 (0.7)	426 (0.5)	131 (0.5)	51 (0.5)	162 (0.6)
40-49	4,848 (4.4)	210 (3.3)	539 (4.2)	2,969 (3.8)	890 (3.6)	396 (3.7)	1,058 (3.8)
50-54	6,002 (5.4)	248 (3.9)	574 (4.4)	3,683 (4.7)	1,229 (5.0)	506 (4.8)	1,204 (4.3)
55-69	37,487 (33.9)	1,999 (31.2)	4,144 (32.0)	25,018 (32.1)	7,395 (30.3)	3,632 (34.3)	9,194 (32.8)
70+ Years	61,194 (55.3)	3,895 (60.8)	7,528 (58.1)	45,491 (58.4)	14,681 (60.2)	5,971 (56.4)	16,267 (58.1)
**Cancer Type According to Site**							
Colon cancer (C18)	69,301 (62.6)	3,993 (62.3)	8,748 (67.5)	50,449 (64.7)	15,723 (64.4)	6,363 (60.1)	17,853 (63.7)
Colon proximal (C18.0, C18.2-C18.5)	34,577 (31.2)	2,097 (32.7)	3,599 (27.8)	24,586 (31.6)	8,222 (33.7)	2,778 (26.3)	9,107 (32.5)
Colon distal (C18.6-C18.7)	28,126 (25.4)	1,617 (25.2)	3,118 (24.1)	19,573 (25.1)	6,158 (25.2)	2,578 (24.4)	7,208 (25.7)
Colon not otherwise specified (C18.8-C18.9)	6,598 (6.0)	279 (4.4)	2,031 (15.7)	6,290 (8.1)	1,343 (5.5)	1,007 (9.5)	1,538 (5.5)
Rectal cancer (C19/C20)	41,421 (37.4)	2,417 (37.7)	4,215 (32.5)	27,478 (35.3)	8,679 (35.6)	4,219 (39.9)	10,152 (36.3)
**Time Period (Year of Diagnosis)**							
2003-2005	21,650 (19.6)	1,484 (23.2)	2,296 (17.7)	16,049 (20.6)	3,768 (15.4)	2,342 (22.1)	5,668 (20.2)
2006-2008	23,940 (21.6)	1,395 (21.8)	2,461 (19.0)	15,801 (20.3)	5,151 (21.1)	2,226 (21.0)	5,694 (20.3)
2009-2011	22,769 (20.6)	1,272 (19.8)	2,816 (21.7)	15,326 (19.7)	5,326 (21.8)	2,030 (19.2)	5,566 (19.9)
2012-2014	21,923 (19.8)	1,189 (18.5)	2,739 (21.1)	15,244 (19.6)	5,233 (21.4)	1,957 (18.5)	5,509 (19.7)
2015-2017	20,440 (18.5)	1,070 (16.7)	2,651 (20.5)	15,507 (19.9)	4,924 (20.2)	2,027 (19.2)	5,568 (19.9)
**Grading**							
Low grade (G1)	5,550 (5.2)	301 (4.9)	887 (7.6)	2,799 (3.8)	2,152 (9.1)	391 (3.8)	1,722 (6.5)
Intermediate (G2)	81,674 (77.1)	4,980 (80.6)	8,181 (70.3)	56,210 (76.1)	16,758 (70.7)	8,157 (80.0)	20,154 (75.8)
High grade (G3/G4)	18,756 (17.7)	897 (14.5)	2,566 (22.1)	14,875 (20.1)	4,804 (20.3)	1,644 (16.1)	4,709 (17.7)
**UICC (Union for International Cancer Control) Stage**							
I	17,638 (15.9)	652 (10.2)	1,083 (8.4)	6,771 (8.7)	2,670 (10.9)	799 (7.6)	3,595 (12.8)
II	22,013 (19.9)	896 (14.0)	1,763 (13.6)	10,207 (13.1)	3,612 (14.8)	998 (9.4)	5,071 (18.1)
III	21,448 (19.4)	967 (15.1)	2,103 (16.2)	12,216 (15.7)	3,491 (14.3)	1,049 (9.9)	5,388 (19.2)
IV	20,399 (18.4)	1,143 (17.8)	2,600 (20.1)	10,318 (13.2)	3,532 (14.5)	1,769 (16.7)	5,137 (18.3)
Unknown	29,224 (26.4)	2,752 (42.9)	5,414 (41.8)	38,415 (49.3)	11,097 (45.5)	5,967 (56.4)	8,814 (31.5)

With the exception of the first rows, DCO (death certificate only)-cases, cases not being histologically confirmed, other than adenocarcinoma and appendiceal cancers have been excluded from the analyses.

Results are presented as absolute (relative) frequencies, unless otherwise indicated.

CRC was slightly more frequent in men than in women. Median age at diagnosis was 72.2 years (25th -75th percentile: 63.7–79.3), with 95% of all cases occurring in individuals at the age of 50 or older. About two-thirds of all CRC were located in the colon. The proportion of unknown cancer stages showed a high variation between the federal states. Overall, 38% of all cases had an unknown stage (range: 26.4–56.4%; Table 1).

### Incidence trends over time according to site, sex and age at diagnosis

Figure 1 shows annual, age-specific incidence rates for the total population and stratified by sex. Incidence trends for women and men showed similar patterns within most of the subgroups defined by tumour site and age group.

In general, after quite stable incidence from 2003 to 2007, incidence decreased since 2008 in the three upper age groups (i.e. 50–54, 55–69 and 70 + years) for all tumour sites. After several years of decline, CRC and colon incidence started to rise again (from 2015 on in the age group 50–54 years and from 2016 on in the age group 70 + years).

In the age group 40–49 years a slight increase of incidence was observed over time, with a decrease for males (and total) for the last two to three years.

In the age group 35–39 years the overall CRC incidence was as low as < 7/100,000. Overall CRC and rectal cancer incidence tended to increase over time.

In the age group 15–34 years the overall CRC incidence rate was even lower with < 2/100,000, but incidence constantly increased over the past 15 years by 4.3% [95% CI: 3.3; 5.5] per year (Table 2).

**Table 2:** Time trends in age-standardized and age-specific colorectal, colon and rectal cancer incidence rates for the total population and according to site, age at diagnosis & stage

Population	Stage	Annual Incidence Rate per 100,000 During Early Screening Phase (2003-2005)	Most Recent Annual Incidence Rate per 100,000 (2015-2017)	Absolute Difference in Rates	Relative Difference in Rates (%)	APC	95% CI
**Colorectal Cancer (C18-C20)**							
**Total Population**	All Stages	41.9	34.9	-7.0	-16.7%	2003-2007: 0.4%	[-1; 1.8]
						2007-2017: -2.2%	[-2.5; -1.8]
	Early Stage	17.4	15.3	-2.0	-11.7%	2003-2017: -1%	[-1.3; -0.7]
	Late Stage	24.5	19.6	-5.0	-20.2%	2003-2006: 1.9%	[-1; 4.8]
						2006-2017: -2.7%	[-3; -2.3]
**15-34 Years**	All Stages	0.9	1.4	0.6	65.7%	2003-2017: 4.3%	[3.3; 5.3]
	Early Stage	0.3	0.5	0.2	77.6%	2003-2017: 5.7%	[3; 8.5]
	Late Stage	0.6	0.9	0.3	59.5%	2003-2017: 3.6%	[2.2; 5]
**35-39 Years**	All Stages	4.7	5.2	0.5	10.1%	2003-2017: 1.4%	[0.2; 2.6]
	Early Stage	1.5	1.8	0.3	20.5%	2003-2017: 2.4%	[0.5; 4.4]
	Late Stage	3.2	3.4	0.2	5.5%	2003-2017: 0.8%	[-0.7; 2.4]
**40-49 Years**	All Stages	14.7	16.2	1.5	10.2%	2003-2017: 0.7%	[0; 1.3]
	Early Stage	5.7	6.1	0.4	7.7%	2003-2017: 0.8%	[0.1; 1.5]
	Late Stage	9.0	10.1	1.0	11.8%	2003-2017: 0.6%	[-0.3; 1.5]
**50-54 Years**	All Stages	42.4	39.6	-2.8	-6.5%	2003-2008: 1.5%	[0.3; 2.7]
						2008-2012: -3.8%	[-6.4; -1.2]
						2012-2017: 0.8%	[-0.4; 2]
	Early Stage	15.7	15.9	0.2	1.3%	2003-2017: -0.1%	[-0.9; 0.7]
	Late Stage	26.6	23.7	-2.9	-11.1%	2003-2008: 1.5%	[-0.9; 4]
						2008-2012: -5.3%	[-10.3; -0.1]
						2012-2015: 2.9%	[-7.6; 14.6]
						2015-2017: -4%	[-13.9; 6.9]
**55-69 Years**	All Stages	129.3	95.7	-33.6	-26.0%	2003-2008: -1.3%	[-2; -0.5]
						2008-2011: -4.6%	[-7.7; -1.3]
						2011-2017: -2.2%	[-2.8; -1.7]
	Early Stage	54.7	40.4	0.2	77.6%	2003-2017: -2.6%	[-2.9; -2.2]
	Late Stage	74.6	55.3	0.3	59.5%	2003-2006: -0.1%	[-3.6; 3.5]
						2006-2017: -3.1%	[-3.6; -2.6]
**70+ Years**	All Stages	262.5	222.2	-40.2	-15.3%	2003-2007: 1.5%	[-0.3; 3.3]
						2007-2017: -2.3%	[-2.7; -1.8]
	Early Stage	109.8	106.4	0.3	20.5%	2003-2007: 1.7%	[-0.9; 4.3]
						2007-2017: -0.9%	[-1.5; -0.2]
	Late Stage	152.7	115.9	0.2	5.5%	2003-2005: 3.6%	[-1.5; 9]
						2005-2008: -1%	[-5.9; 4.2]
						2008-2017: -3.5%	[-3.9; -3]
**Colon Cancer (C18)**							
**Total Population**	All Stages	25.8	21.7	-4.1	-15.9%	2003-2007: 0.1%	[-1.6; 1.8]
						2007-2017: -2%	[-2.4; -1.6]
	Early Stage	10.8	10.2	-0.5	-5.0%	2003-2017: -0.4%	[-0.8; -0.1]
	Late Stage	15.0	11.5	-3.6	-23.7%	2003-2006: 0.9%	[-1.9; 3.8]
						2006-2017: -2.9%	[-3.3; -2.6]
**15-34 Years**	All Stages	0.5	0.8	0.3	47.8%	2003-2017: 3.3%	[2.1; 4.5]
	Early Stage	0.2	0.3	0.1	52.6%	2003-2017: 4.2%	[0.7; 7.8]
	Late Stage	0.4	0.5	0.2	44.6%	2003-2017: 2.7%	[0.9; 4.6]
**35-39 Years**	All Stages	2.9	2.6	-0.3	-10.2%	2003-2017: 0.3%	[-1.7; 2.5]
	Early Stage	0.9	0.9	0.0	2.6%	2003-2017: 0.3%	[-1.7; 2.5]
	Late Stage	2.0	1.7	-0.3	-15.7%	2003-2017: -0.6%	[-2.6; 1.5]
**40-49 Years**	All Stages	7.9	8.7	0.9	10.9%	2003-2017: 0.6%	[-0.1; 1.4]
	Early Stage	3.1	3.5	0.4	13.9%	2003-2017: 1.1%	[0.3; 2]
	Late Stage	4.8	5.2	0.4	9.3%	2003-2017: 0.3%	[-0.9; 1.4]
**50-54 Years**	All Stages	22.5	21.3	-1.3	-5.7%	2003-2009: 0.8%	[-0.5; 2]
						2009-2012: -6.3%	[-12.9; 0.8]
						2012-2017: 2.7%	[1.1; 4.4]
	Early Stage	8.4	9.6	1.2	13.9%	2003-2005: -6.9%	[-20.1; 8.4]
						2005-2008: 7.9%	[-7.3; 25.7]
						2008-2015: -2.5%	[-5; 0.1]
						2015-2017: 15.7%	[-0.6; 34.8]
	Late Stage	14.2	11.7	-2.4	-17.2%	2003-2008: 0.6%	[-3.2; 4.5]
						2008-2012: -7.1%	[-14.8; 1.2]
						2012-2015: 5.6%	[-11; 25.3]
						2015-2017: -6.2%	[-20.9; 11.4]
**55-69 Years**	All Stages	75.7	56.0	-19.7	-26.0%	2003-2008: -1.6%	[-2.7; -0.5]
						2008-2011: -4.5%	[-9.1; 0.4]
						2011-2017: -2%	[-2.9; -1.2]
	Early Stage	32.4	25.6	0.4	13.9%	2003-2017: -2%	[-2.3; -1.7]
	Late Stage	43.3	30.4	0.4	9.3%	2003-2006: -0.5%	[-3.6; 2.7]
						2006-2012: -4.3%	[-5.7; -3]
						2012-2017: -2.1%	[-3.5; -0.7]
**70+ Years**	All Stages	178.8	155.1	-23.6	-13.2%	2003-2006: 2.3%	[-1.1; 5.9]
						2006-2017: -1.8%	[-2.3; -1.4]
	Early Stage	74.9	77.6	0.4	13.9%	2003-2006: 2.5%	[-1.8; 7]
						2006-2017: -0.1%	[-0.7; 0.5]
	Late Stage	103.8	77.5	0.4	9.3%	2003-2006: 2.4%	[-1.1; 6]
						2006-2017: -3.3%	[-3.8; -2.9]
**Rectal Cancer (C19/C20)**							
**Total Population**	All Stages	16.1	13.2	-2.9	-18.0%	2003-2007: 1%	[-0.8; 2.8]
						2007-2017: -2.5%	[-2.9; -2.1]
	Early Stage	6.6	5.1	-1.5	-22.7%	2003-2017: -2.1%	[-2.5; -1.7]
	Late Stage	9.5	8.1	-1.4	-14.8%	2003-2008: 1.3%	[-0.2; 2.7]
						2008-2017: -2.7%	[-3.2; -2.1]
**15-34 Years**	All Stages	0.3	0.6	0.3	97.1%	2003-2017: 5.9%	[4.4; 7.5]
	Early Stage	0.1	0.2	0.1	121.9%	2003-2017: 7.6%	[4.4; 10.9]
	Late Stage	0.2	0.4	0.2	84.5%	2003-2017: 5%	[3; 7.1]
**35-39 Years**	All Stages	1.9	2.6	0.8	41.5%	2003-2017: 2.7%	[0.7; 4.7]
	Early Stage	0.6	0.9	0.3	46.4%	2003-2017: 2.7%	[0.7; 4.7]
	Late Stage	1.3	1.7	0.5	39.1%	2003-2017: 2.8%	[0.9; 4.7]
**40-49 Years**	All Stages	6.8	7.5	0.6	9.2%	2003-2017: 0.7%	[0; 1.4]
	Early Stage	2.6	2.6	0.0	0.4%	2003-2017: 0.4%	[-0.5; 1.3]
	Late Stage	4.3	4.9	0.6	14.5%	2003-2017: 0.9%	[-0.1; 1.9]
**50-54 Years**	All Stages	19.8	18.4	-1.5	-7.5%	2003-2007: 2.2%	[-0.7; 5.1]
						2007-2017: -1.7%	[-2.4; -1]
	Early Stage	7.3	6.4	-1.0	-13.2%	2003-2017: -1.1%	[-2; -0.2]
	Late Stage	12.5	12.0	-0.5	-4.2%	2003-2017: -0.7%	[-1.5; 0]
**55-69 Years**	All Stages	53.7	39.7	-13.9	-26.0%	2003-2007: -0.7%	[-2.9; 1.6]
						2007-2017: -3.2%	[-3.7; -2.6]
	Early Stage	22.3	14.8	0.0	0.4%	2003-2017: -3.4%	[-3.9; -2.9]
	Late Stage	31.4	24.9	0.6	14.5%	2003-2007: 0.7%	[-1.9; 3.4]
						2007-2017: -2.9%	[-3.5; -2.3]
**70+ Years**	All Stages	83.7	67.1	-16.6	-19.9%	2003-2008: 1.2%	[-0.3; 2.7]
						2008-2017: -3.3%	[-3.8; -2.7]
	Early Stage	34.9	28.7	0.0	0.4%	2003-2007: 1.5%	[-1.6; 4.8]
						2007-2017: -2.5%	[-3.3; -1.8]
	Late Stage	48.8	38.4	0.6	14.5%	2003-2008: 1.6%	[-0.1; 3.3]
						2008-2017: -3.7%	[-4.3; -3]

Age-standardized and age-specific incidence rates = Cases per 100,000;

Incidence Rates for the Total Population are given as age-standardized rate using the European Standard (1976);

Early Stage = UICC (Union for International Cancer Control) I & II, Late Stage = UICC III & IV;

APC = Annual percent changes;

CI = Confidence interval

With the exception of the age group 40–49 years, all trends were more pronounced in rectal than in colon cancer.

### Stage distribution of colorectal, colon and rectal cancer according to time of diagnosis and age at diagnosis

**Fig. 2:**
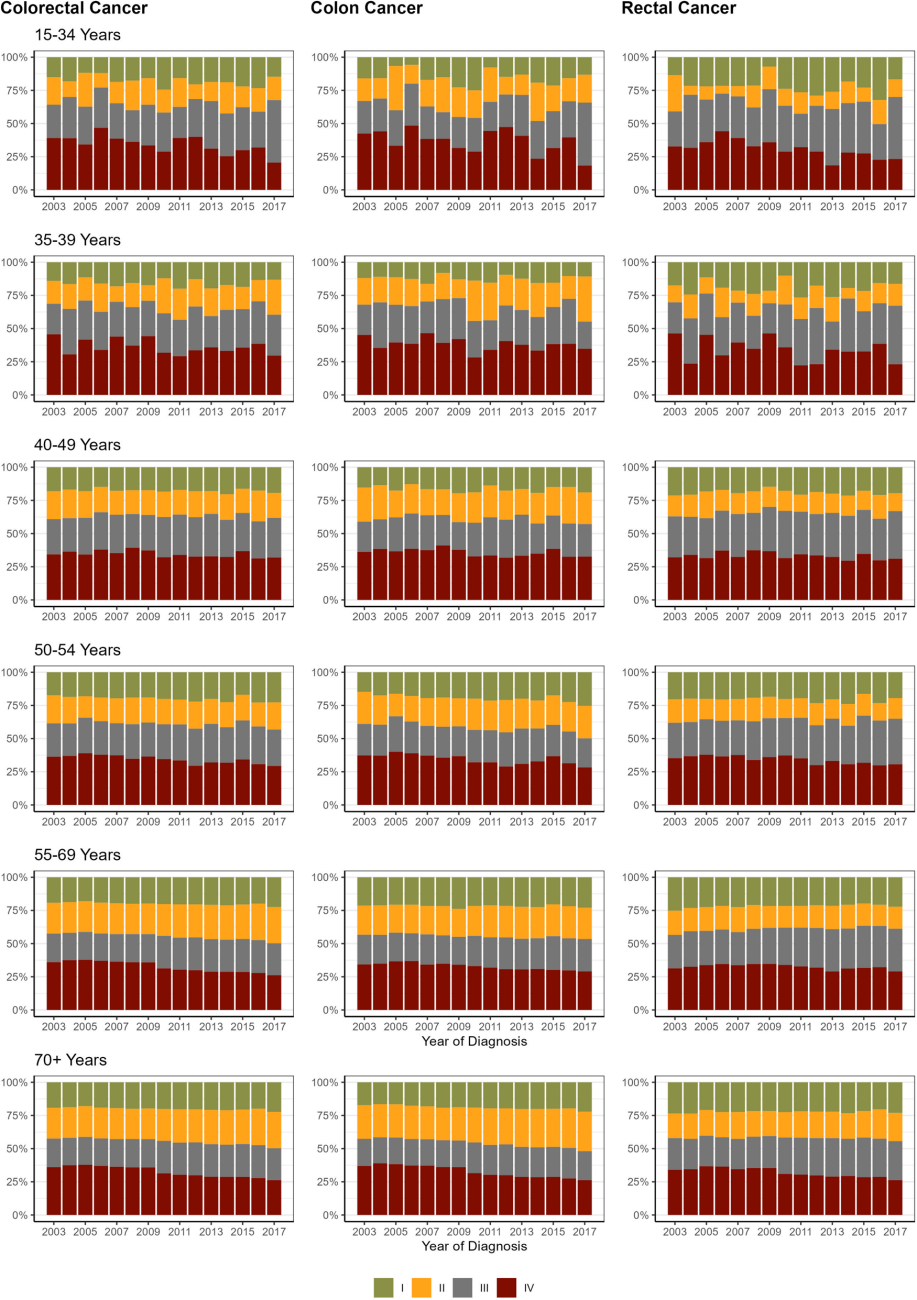
Time Trends of Tumour Stage Distribution (UICC (Union for International Cancer Control) stages) According to Site & Age at Diagnosis. UICC (Union for International Cancer Control) stage distribution after imputation of missing stage information (38%, method multiple imputation with chained equations (mice), 5 imputations, 25 iterations)

After imputation of missing stages, a general pattern could be observed in the two oldest age groups (55–69, 70+; Fig. 2): Overall, more than 55% of all CRC cases were late stage tumours (stage III or IV; age group 55–69: 57.8%, age group 70+: 55.3%). Tumours with stage IV were most frequently diagnosed over the whole time period, but the proportion decreased over time (age group 55–69, years 2003 to 2017 CRC: 33.1 to 29.1%; colon: 34.3 to 29.1%; rectum: 31.4 to 29.1%; age group 70+, 2003 to 2017 CRC: 36.0 to 26.2%; colon: 37.0 to 26.2%; rectum: 34.0 to 26.2%). At the end of the study period, i.e. in 2017, the proportion of early stage colon cancers (stage I or stage II) in the age group 70 + was 51.9% (early stage rectal cancer: 44.3%). The proportion of stage II tumours in colon cancer (29.8%) was slightly higher than that of stage IV (26.2%), which was not the case for rectal cancer (stage II: 21.2%, stage IV: 26.2%). In the age group 55–69 the proportion of late stage tumours remained high for colon (53.5% at the end of the study period) as well as for rectum cancer (61.2%).

Patterns in the age group 50–54 were quite similar and overall the proportion of late stage CRC exceeded 50% as well. In 2017, early stage tumours (stage I and stage II) made up 49.8% of all colon cancers (rectal: 35.0%, CRC: 43.2%). In the age group 40–49 the stage distribution over time was quite stable. But in rectal cancer late stage tumours slightly increased over time. Due to low annual numbers of incident cases (see Supplemental Table 1), the stage distributions in the two youngest age groups showed higher variations year by year, but stayed more or less the same over time.

In late stage rectal cancer, a shift was observed from UICC stage IV to stage III being the most prevalent stage. This shift was observed in all age groups.

### Incidence trends over time according to site, stage and age at diagnosis

In general, the overall incidence rates of colorectal, colon and rectal cancer stratified by sex (Supplemental Table 2) and stratified by sex and age groups (Supplemental Table 1) decreased over time.

In the total population, a relative difference (RD) of -16.7% was observed for CRC incidence rates between the early screening phase (2003–2005) and the most recent time period (2015–2017; Table 2). The relative difference between the rates was slightly greater for rectal (RD: -18%) than for colon cancer (RD: -15.9%). Significant declines (APC of about − 2.0 to -2.5%) were found for the time period 2006/2007–2017 for all tumour sites considered.

When stage was further considered, the trends differed between colon and rectal cancer. In colon cancer the changes were more pronounced in late stage (RD: -23.7%; APC 2006–2017: -2.9%) than in early stage tumours (RD: -5%; APC 2003–2017: -0.4%). In rectal cancer, however, the changes were greater for early stage tumours (RD: -22.7%; APC 2003–2017: -2.1% vs. late stage RD: -14.8%; APC 2008–2017: -2.7%; Table 2).

**Fig. 3:**
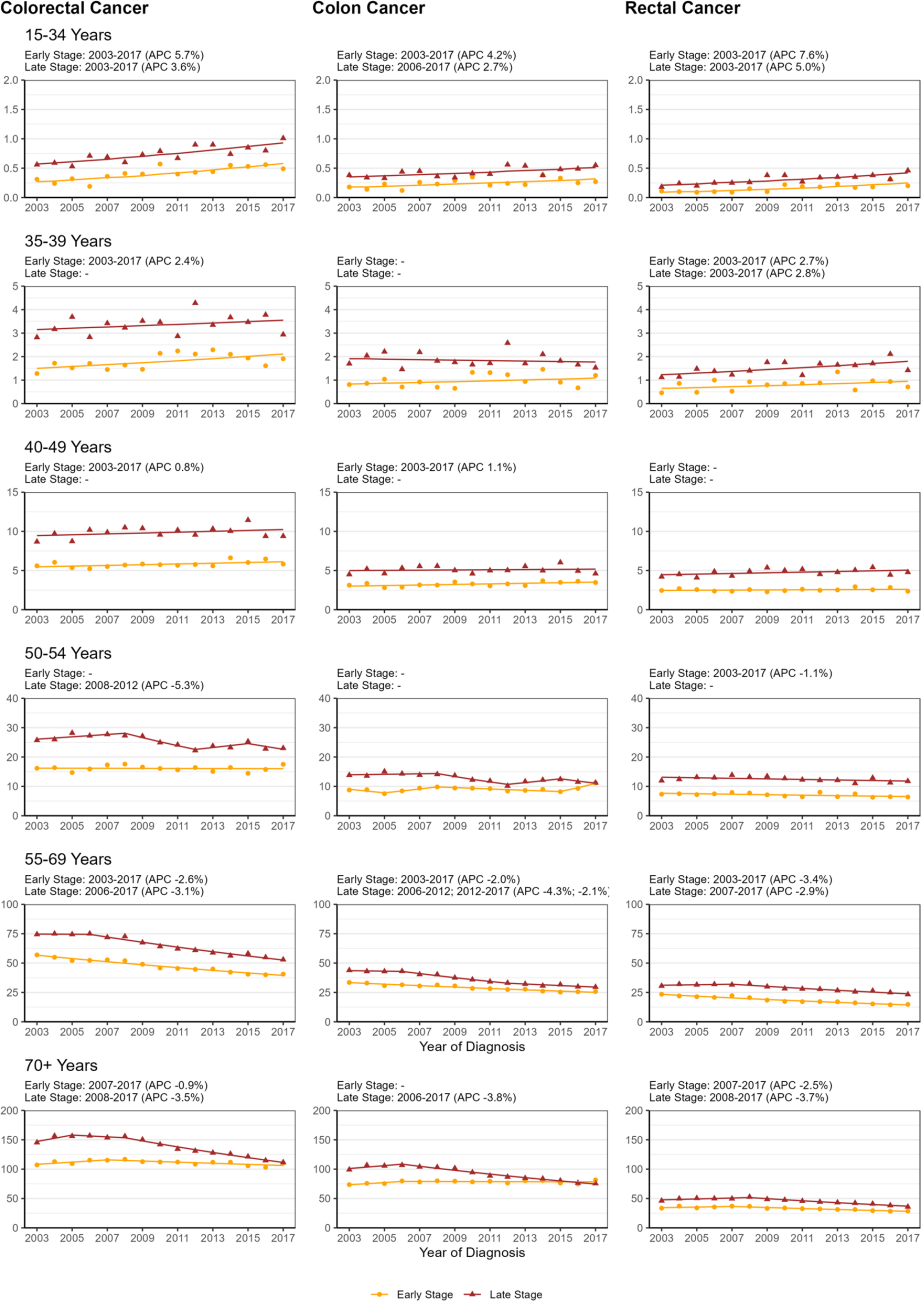
Time Trends of Age-Specific Incidence Rates (Cases per 100,000) According to Site, Age at Diagnosis & Stage. Age-specific incidence rates = Cases per 100,000; Displayed are Crude Observations and Regression Lines; Above each panel time periods with significant incidence trends plus corresponding annual percent changes (APC) are indicated by stage; Yellow Circles = Early Stage (UICC (Union for International Cancer Control) I & II), Red Triangles = Late Stage (UICC III & IV)

 Figure 3 displays incidence trends by stage, site and age groups. In the oldest age group, significant incidence declines could be observed since 2006 in colon and since 2008 in rectal cancer and in CRC with an APC of about − 3.5% for late stage tumours, in case of rectal cancer also for early stage tumours, and to a lesser extent also for early stage CRC tumours (APC: -0.9%). In the age group 55–69, significant incidence trends were observed for all tumour site by stage combinations (range of APCs: -2.0 to -4.3%). In the age group 50–54 years, significant trends were only observed for late stage CRC (APC 2008–2012: -5.3%) and early stage rectal cancer incidence (APC 2003–2013: -1.1%). In the age group 40–49 years, a significant increase in early stage CRC incidence (APC 2003-2017: 0.8% per year) was observed, mainly due to the increase in colon cancer. In the age group 35–39 years, incidence increases were more pronounced and were significant for early stage CRC (APC 2003–2017: 2.4%), early stage rectal cancer (APC 2003–2017: 2.7%) and late stage rectal cancer (APC 2003–2017: 2.8%). In the youngest age group, increases were even more pronounced, with higher APCs for early stage cancers than for late stage cancers (CRC APCs: 5.7% vs. 3.6%; colon cancer APCs: 4.2% vs. 2.7%; rectal cancer APCs: 7.6% vs. 5.0%).

## Discussion

Our analyses are based on 271,011 adenocarcinomas located in the colorectum and diagnosed between 2003 and 2017. The majority of these CRC are observed in individuals aged 55 years or older and are located in the colon. Therefore, overall trends for CRC incidence are mainly dominated by trends observed for the older age groups and trends for the tumours in the colon. To analyse effects of age and localisation of CRC stratified analyses are needed.

### Falling incidence rates in individuals aged 50 years and older

Our finding of decreasing incidence rates for CRC in total as well as for colon and rectal cancer separately in persons aged 55 or older is in line with the existing literature describing falling incidence rates for countries with a high human development index [1, 4–6, 8–10]. The recent steep decrease observed in regions such as Europe, North America, Oceania, and some countries in Asia is accounted for primarily by CRC screening [4, 5, 7, 9]. Only recently, Bretthauer and colleagues published results on the effects of (invitation to) colonoscopy screening on risks of CRC and related death based on a pragmatic, randomized controlled trial. Based on data from about 84,500 individuals with a median follow-up of 10 years they were able to show that an invitation to CRC screening compared to no invitation reduced the risk for CRC by 18% (risk ratio: 0.82; 95% CI: 0.70–0.93) and the risk of death from CRC was reduced by 10% (risk ratio: 0.90; 95% CI: 0.64 to 1.16) in the intention-to-treat analysis. In the per-protocol analysis comparing the group with actual screening with the group without screening invitation the risk ratio reduced down to 0.69 (95% CI: 0.55–0.83) for CRC diagnosis and to 0.50 (95% CI: 0.27 to 0.77) for death from CRC [22].

In Germany, the national CRC screening programme was launched in 2002 and was provided until 2017 with annual stool tests for the age group 50–54 and two colonoscopies from the age of 55 years on. In the year 2018, the screening modalities were slightly changed and an invitation to the screening was added [24]. Until now, no comprehensive CRC screening evaluation report is available for Germany. Instead, participation rates have been estimated based on a single health insurance (AOK) [37], billing data of the statutory health insurance companies (ZI) [38], or on surveys [39]. It is estimated that approximately 2.5% of men and 2.7% of women in the age group 55–64 undergo a colonoscopy each year, and 26% and 27% respectively perform a FOBT. When a ten-year period is considered the overall participation rate in CRC screening (either with one colonoscopy or three times FOBT performed) increases up to 35% for males and 47% for women aged 55–64. During the period 2009 to 2018 participation rates remain quite stable (colonoscopy) or tend to decrease (-7% for FOBT in women; Suppl. Figure 3) [38]. The observation that participation rates differ between men and women fits well into the general observation that women compared to men as well as individuals with a healthy life-style and higher health literacy are more likely to participate in early detection programs [40]. Explicit reasons for the low participation rates in CRC screening in Germany remain yet unknown. Potential reasons might be a lack of knowledge regarding screening measures, the fear of receiving a “test-positive” result / cancer diagnosis [40], the unpleasant associations and the potential risks that are associated with a colonoscopy, and finally the fact that there is a high number of diagnostic colonoscopies in Germany. One diagnostic colonoscopy is performed per one screening colonoscopy [41] which would lead to an overall colonoscopy rate of clearly over 50% for Germany.

Only six years after the implementation of CRC screening in Germany, the CRC incidence started to decline in screening-entitled age groups. Comparing the CRC incidence rate of the most recent years to that of the early screening phase the relative difference was about − 26% in rectal and colon cancer for the age group 55–69 and somewhat lower in the oldest age group (rectal cancer RD: -20%, colon cancer RD: -13%).

The research group of Hermann Brenner has published several scientific publications investigating the effect of screening colonoscopies on the epidemiology of CRC in Germany [5, 7, 26]. Among them are results from the ESTHER study in which the study participants were compared with CRC cases documented at the Saarland cancer registry. This study showed that participation in colonoscopy screening is associated with a significant risk reduction of CRC diagnosis (adjusted hazard ratio of 0.44) [26]. This risk reduction is slightly higher than the ones reported from the recent RCT which were either based on the intention-to-treat analysis (comparison of receiving a screening invitation vs. no invitation) or based on the per-protocol analysis (comparison of actual screening vs. no invitation) [22]. A current evaluation of population-based data on CRC incidence from Brenner’s working group adds further evidence that screening has contributed to significant reductions in the incidence of distal colon cancer. However, the youngest age group in their report was defined as < 55 years [7].

### Rising incidence rates in individuals younger than 50 years

In our population less than 5% of CRC occurred in individuals younger than 50 years of age (< 55 years: 10%). This and the finding of rising incidence rates among the German population younger than 50 years of age, fit well to the existing evidence, where similar patterns have been described [4–7, 9–19, 42].

When the group of young adults was further differentiated in three age groups, largest increases in CRC incidences were found for the youngest age group (15–34 years; colon: 48%, rectum: 97%), followed by the age groups 35–39 years (increase only in rectal cancer: 41.5%) and 40–49 years (colon: 11%, rectum: 9%). Amongst others Chambers et al. reported that the CRC incidence rise is steepest among persons aged 20–39 [6, 12, 16, 19] and that it is mainly due to a rise in distal colon cancer [16], while others attributed to rise predominantly to proximal colon cancer [19], colon cancer (distal and proximal combined) [6] or rectal cancer [12].

Numerous authors explained the incidence rise of CRC in young adults in North America, the United Kingdom and Europe as birth cohort effects with large increases from the mid-1960s on [6, 8, 16]. Unfortunately, the time period covered in our data is not long enough to add further evidence to this.

Extensive research over the past few decades has delved into the risk factors associated with CRC in older adults. These risk factors are recognized to be multifactorial, both inherited and acquired, and include alcohol consumption, high consumption of processed meat and of red med, diabetes type 2, obesity, physical inactivity, and smoking [13, 15, 43]. Even though the risk factors mentioned above are relevant to both colon and rectal cancer, certain factors are specifically linked to colon cancer [6, 44]. Saad el Din et al. highlighted the necessity for further research into the underlying causes of the differing risks between colon and rectal cancers [12]. Siegel et al. further pointed out that the link between CRC and established risk factors is largely grounded on research in older age groups [10].

Today, it is well known that CRC in younger and in older adults differ with respect to clinical and molecular features such as microsatellite instability or specific gene mutations [16]. However, the increase in CRC in young adults is still not well-understood [15]. Most CRC in young adults occur sporadically and it is believed that the risk factors are largely similar to those linked with CRC in older adults [15].

The rising incidence of CRC in young adults coincides with the increasing rates of obesity and diabetes type 2 in Germany. It is acknowledged that early-life (i.e. childhood and young adulthood) obesity is associated with an elevated risk of developing CRC. And according to Siegel and colleagues, the rising prevalence rates of obesity and type 2 diabetes observed over the past thirty years could potentially explain the rise in CRC incidence among young adults [10]. In Germany, the prevalence of obesity among male adults aged 25–34 years has increased from about 11% in 1990–1992 to 17% in 2008–2011 and in females of the same age from about 9–14% [45], while the prevalence of obesity in children (6% in children aged 3–17 years) has been stable over the last decades [46]. Further, the prevalence of diagnosed diabetes type 2 in the total German population has increased over the last decades (1997–1997: 5.2%; 2008–2011: 7.2%) and has been recently estimated to be about 1% in adults aged 30–34 years and about 3% in adults aged 35–39 years [47, 48]. However, the age- and sex-standardized prevalence of total diabetes (diagnosed, undiagnosed, type 1 and 2) remained stable between 1997-1999 at 9.3% and 2008–2011 at 9.2% [48].

### Young adults are more likely to be diagnosed with late stage tumours

Compared to persons aged 50 and older, patients under the age of 50 are more likely to be diagnosed with more advanced tumour stages regardless of tumour localization (2003–2017 late stage CRC in individuals < 50 years of age: 63.4% vs. 56.5% in individuals 50 + years). Moeller et al. compared patients with CRC younger than 55 years and 55 + years and found a similar, but less pronounced pattern [42]. Petersson et al. reported that about 66% of all colon and about 61% of all rectal tumours in persons younger than 50 years were classified as stage III or IV [13]. We observed high proportions of late stage tumours as well, but with lower proportions for colon (< 50 years: 60.6% vs. 50+: 51.5%) than for rectal cancer (< 50 years: 65.2% vs. 50+: 59.9%) in the most recent time period as well as for the early screening period (colon < 50 years: 62.3% vs. 50+: 57.9%; rectum < 50 years: 63.2% vs. 50+: 58.7%).

### Possible reasons for trends in stage-specific incidence rates

As described above, there was a marked heterogeneity in the incidence trends between CRC in younger and in older adults. These changes in CRC incidence in Germany have been recently predicted via means of Markov models [25] and are further in line with the existing literature [15, 19, 42].

The significant decline in late stage CRC incidence of screening-entitled, older adults, which started about 6 years after the implementation of the national CRC screening programme, is promising and can be – considering our study design – cautiously interpreted as a success of the screening activities in Germany. Also, the decline in early stage CRC is likely linked to the colonoscopy screening, as it has been proven that colonoscopy with polypectomy will also reduce the incidence of early stage CRC [21].

As done above, it can be discussed whether etiological factors only led to the increase of CRC in young adults or whether an increased awareness or data artefacts contributed to this finding as well. On the one hand, an increase in CRC in young adults has been described for a substantial number of countries with a high human development index or a westernized life-style [4–7, 9, 11–19, 42], making it highly likely that etiological factors contributed to the trend as well. On the other hand, for many years the Burda Foundation has been running massive awareness campaigns for CRC screening in Germany. These campaigns include advertisements in newspapers, radio and TV spots in Germany. It has to be mentioned that these campaigns were not specifically aimed at the target group of the colonoscopy screening, but deliberately addressed younger people as well, which might have led to increased awareness in younger individuals, too. Thus, younger individuals are today more likely to seek a gastroenterologist when symptoms occur or when family members are confronted with a CRC diagnosis. This might especially explain the significant increases in early stage CRC incidence in all younger age groups and the increases for early and late stage incidence in rectal cancer in adults up to 39 years of age.

### Strengths and limitations

Our analysis is based on a high number of real-world single-patient data on CRC incidence in Germany, and not on estimations or statistical modelling. We used population-based data from seven selected population-based cancer registries. The selection of cancer registries was not at random. Their respective regions include about 36% of the German population. The respective cancer registries offer a consistently high level of completeness, a low DCO-rate and operate for at least for 10 years. All of that reduces possible effects of cancer registration on incidence trends. Using a large data set consisting of 271,011 cases, we were able to describe trends also in individuals younger age groups with a sufficient sample size.

A limitation of the analysis lies in high number of missing information on tumour stage. Therefore, we used multiple imputation to derive stage for all CRC. It has also to be mentioned that the classification systems for stage changed of time (from TNM 4 to 8), but the impact on trends should be small, especially after stratification into two stage groups (early and late stage). And finally, the federal status of Bavaria and Lower Saxony seem to have slight underreporting of CRC cases for the year 2003, which may lead to a slight underestimation of CRC incidence for the early screening phase (see Additional File 2). And finally, the incidence data provided by the Centre for Cancer Registry Data (ZfKD) included only data from 2003 on, which did not allow to describe incidence before the start of the national screening program. However, the authors have described baseline incidence rates and early effects of the screening program in previous publications [28, 49].

## Conclusion

The national CRC screening programme was launched in 2002 in Germany. The decline in CRC incidence, especially of late stage cancers, in screening-eligible individuals at the age of 50 or older started about six years after the introduction. This decline is in line with the expected effects of the screening programme. The increase in CRC incidence in adults aged 50–54 and 70 + observed from 2015/2016 on warrants further observation of CRC epidemiology.

CRC incidence in younger individuals – who are not screening-entitled – increased during the same time period. Interestingly, early stage CRC rose stronger than late stage CRC in the younger age groups, indicating raised awareness and/or opportunistic screening. Given that the age-specific incidence rates in individuals at the age of 35 to 39 years and at the age of 40 to 49 years equal only 4.5% and 9%, respectively, of that in individuals at the age of 55 or older, it is questionable whether costs and harms of population-based CRC screening in young adults would outweigh the benefits.

### Supplementary Information

**Additional file 1. Supplemental Figure 1:** Map of Germany Indicating the Regions/Cancer Registries That Contributed Data to the Pooled Data Set.

**Additional file 2. Supplemental File 1:** Histologic/Anatomic Site Coding.

**Additional file 3. Supplemental Table 1:** Number of Incident Cases and Age-Specific Incidence Rates of Colorectal, Colon and Rectal Cancer by Sex & Age at Diagnosis and by Sex, Age & Period of Diagnosis.

**Additional file 4. Supplemental File 2:** Joinpoint Regression Model Specifications.

**Additional file 5. Supplemental Figure 2:** Total Number of Incident Cases by Federal State/Cancer Registry & Year of Diagnosis.

**Additional file 6. Supplemental Figure 3:** Annual CRC Screening Participation Rates by Sex and Year According to Steffen et al. 2020.

## Data Availability

The data sets generated and analysed during the current study are not publicly available due to the data usage agreement of the Centre of Cancer Registry Data which prohibits the disclosure of data to third parties. But data is freely available from the Centre of Cancer Registry Data at the Robert Koch Institute upon reasonable request as regulated by the Law on the Merging of Cancer Registry Data (as of 18th August 2021) § 4 (2). Official request can be made via email to antrag-krebsdaten@rki.de (for details on the request refer to https://www.krebsdaten.de/Krebs/EN/Content/ScientificUseFile/scientificusefile_node.html).

## Abbreviations

ASR: Age–standardized rate
CRC: Colorectal cancer
CT: Computed tomography
DCO: Death certificate only
gFOBT: Guaiac based faecal occult blood test
iFOBT: Immunological faecal occult blood test
UICC: Union for International Cancer Control
ZfKD: Zentrum für Krebsregisterdaten / Centre for Cancer Registry Data

## Declarations

After reviewing the study protocol, the scientific committee of the Centre for Cancer Registry Data grants permission to researches to use the (anonymized) nationwide data set. The permission to use the data set was granted on 25th June 2021 under file number “5.03.04/0002#0083 − 0003” by the scientific committee of the Centre for Cancer Registry data.

Research with anonymous data without patient contact was not subject to an ethics vote in Germany at the time of study protocol generation in 2021. However, ethical approval was subsequently obtained from the ethics committee of the University of Luebeck (31st Mai 2023; file number 2023 − 467).

All methods were carried out in accordance with relevant guidelines and regulations such as the Declaration of Helsinki and Guideline for Good Epidemiological Practise.
