# Supplementary figures and images for: Temporal trends in age- and stage-specific incidence of colorectal adenocarcinomas in Germany
Source: BMC Cancer. 2023 Dec 1;23:1180. doi: 10.1186/s12885-023-11660-1 (PMC10693075; doi:10.1186/s12885-023-11660-1)

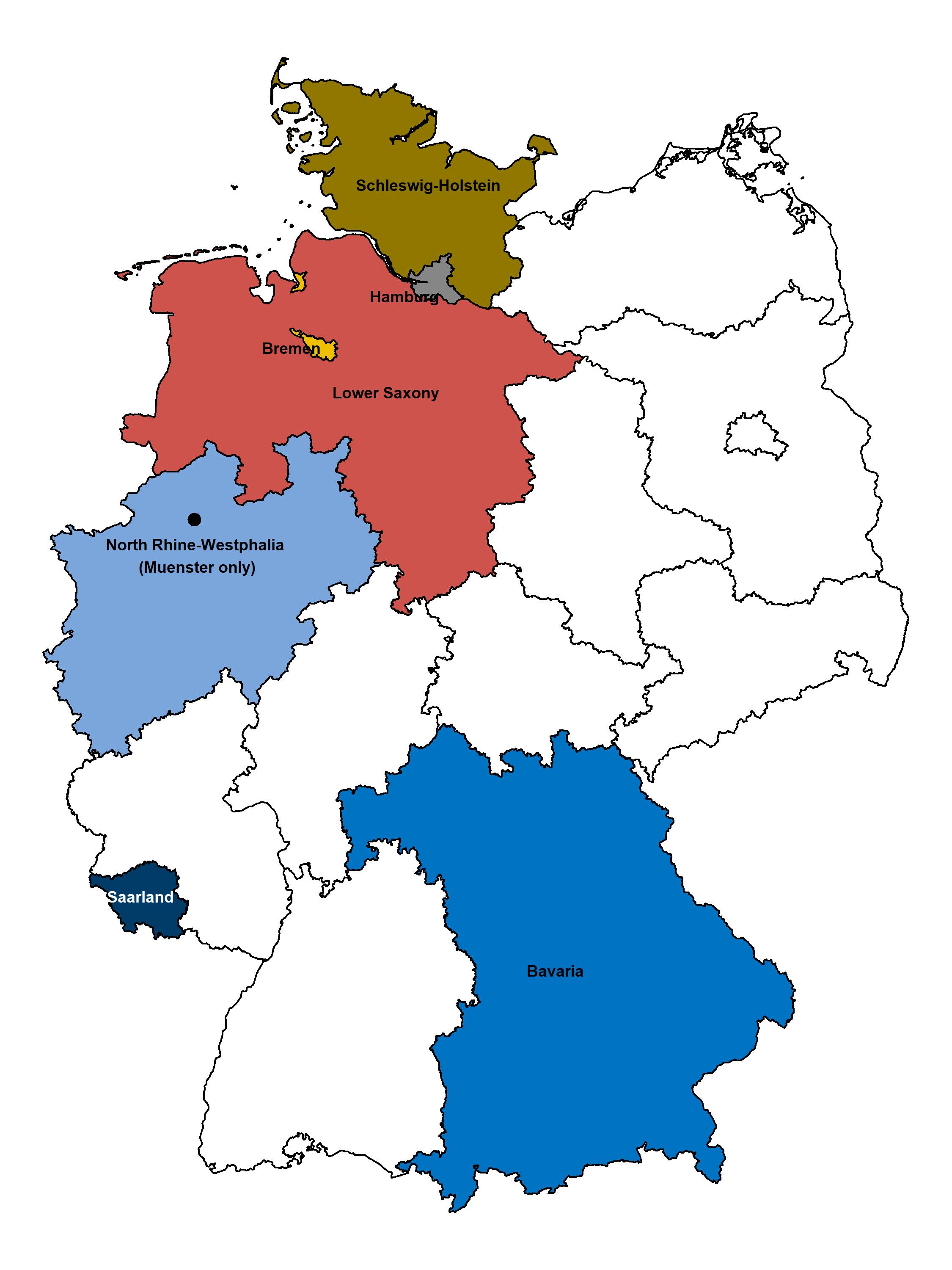

Supplement: Supplementary file 1 — Additional file 1. Supplemental Figure 1: Map of Germany Indicating the Regions/Cancer Registries That Contributed Data to the Pooled Data Set. [file 12885_2023_11660_MOESM1_ESM.jpg]

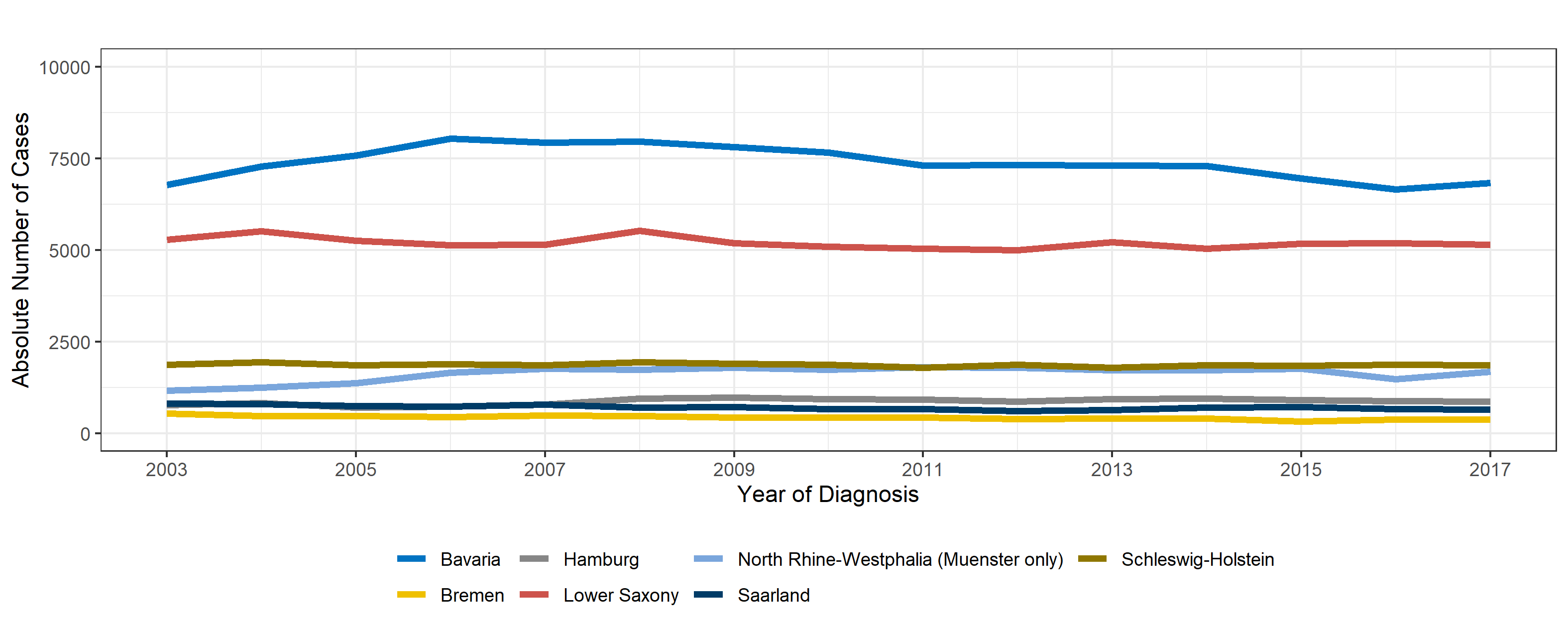

Supplement: Supplementary file 5 — Additional file 5. Supplemental Figure 2: Total Number of Incident Cases by Federal State/Cancer Registry & Year of Diagnosis. [file 12885_2023_11660_MOESM5_ESM.png]

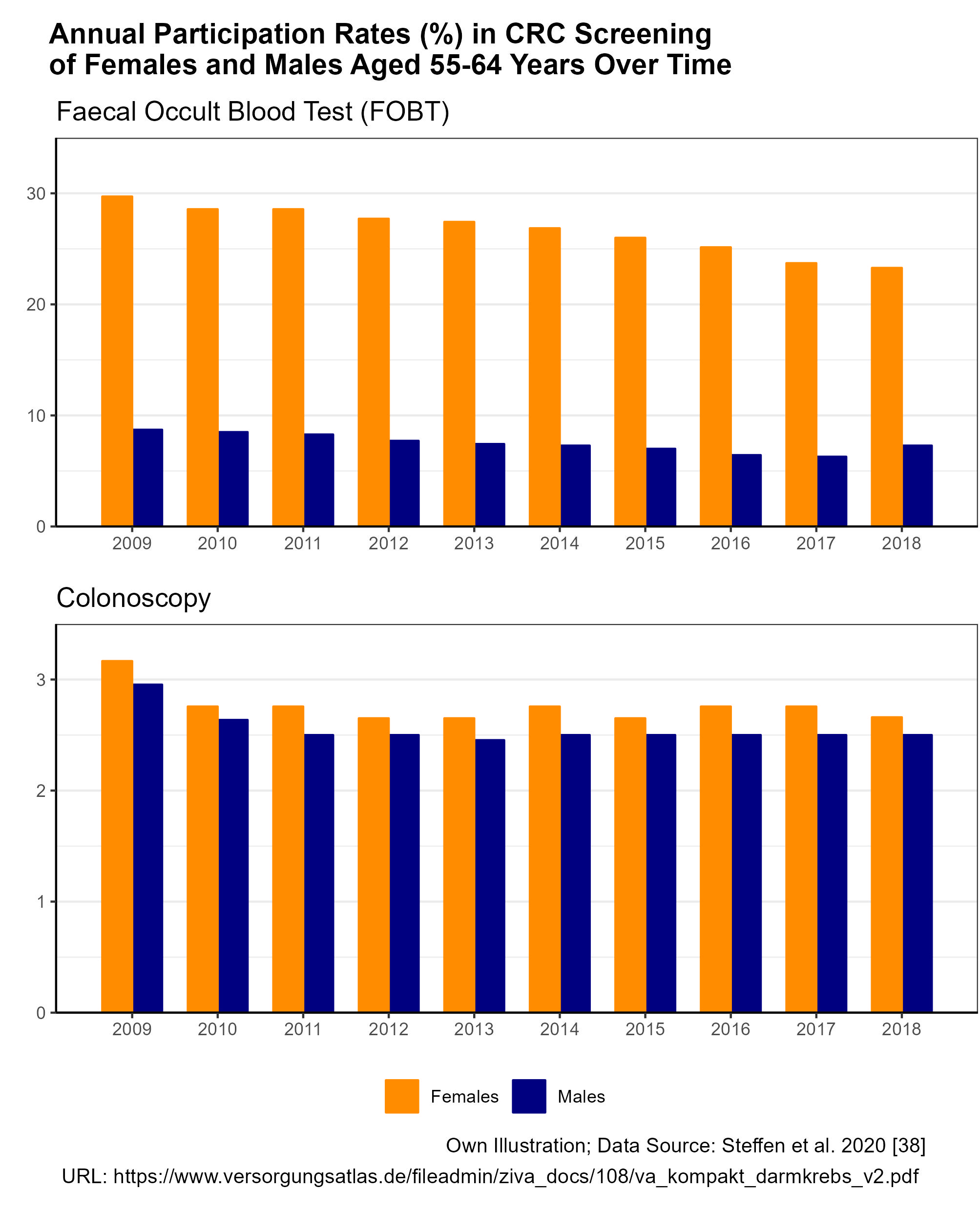

Supplement: Supplementary file 6 — Additional file 6. Supplemental Figure 3: Annual CRC Screening Participation Rates by Sex and Year According to Steffen et al. 2020. [file 12885_2023_11660_MOESM6_ESM.jpg]
