# Supplementary material for: Temporal trends in age- and stage-specific incidence of colorectal adenocarcinomas in Germany
Source: BMC Cancer. 2023 Dec 1;23:1180. doi: 10.1186/s12885-023-11660-1 (PMC10693075; doi:10.1186/s12885-023-11660-1)
Supplement: Supplementary file 2 — Additional file 2. Supplemental File 1: Histologic/Anatomic Site Coding. [file 12885_2023_11660_MOESM2_ESM.docx]

***Supplemental File 1:*** *Histologic/Anatomic Site Coding*

ICD-O-3 histology and behaviour codes were used to define invasive adenocarcinomas.

We included

(i) Behaviour code = 3 determined invasive neoplasm.

(ii) Adenocarcinoma was defined as histology codes 8140, 8141, 8143, 8144, 8210,

8211, 8213, 8220, 8221, 8260-8265, 8255, 8260-8263, 8310, 8323, 8440, 8460,

8470, 8472, 8480-8482, 8570, 8574, 8576.

Tumours with other histology or other behaviour codes were excluded.

ICD-O-3 topographic (anatomic site) codes were used to define colon and rectal cancer.

As defined in Fritz A, Percy C, Jack A, et al. Eds., International Classification of Diseases for

Oncology, 3rd ed., Geneva: World Health Organization, 2000.

We included

(i) Colon cancer: C18.0, C18.2-C18.9

(ii) Rectal cancer: C19.9, C20.9

We excluded appendiceal cancers (C18.1).
