# Supplementary material for: Temporal trends in age- and stage-specific incidence of colorectal adenocarcinomas in Germany
Source: BMC Cancer. 2023 Dec 1;23:1180. doi: 10.1186/s12885-023-11660-1 (PMC10693075; doi:10.1186/s12885-023-11660-1)
Supplement: Supplementary file 3 — Additional file 3. Supplemental Table 1: Number of Incident Cases and Age-Specific Incidence Rates of Colorectal, Colon and Rectal Cancer by Sex & Age at Diagnosis and by Sex, Age & Period of Diagnosis. [file 12885_2023_11660_MOESM3_ESM.docx]

***Supplemental Figure 1:*** *Map of Germany Indicating the Regions/Cancer Registries That Contributed Data to the Pooled Data Set*

***Supplemental Figure 2:*** *Total Number of Incident Cases by Federal State/Cancer Registry & Year of Diagnosis*

***Supplemental Figure 3:*** *Annual* *CRC Screening Participation Rates by Sex & Year According to Steffen et al. 2020*

***Supplemental Table 1:*** *Number of Incident Cases and Age-Specific Incidence Rates of Colorectal, Colon and Rectal Cancer by Sex & Age at Diagnosis and by Sex, Age & Period of Diagnosis*

|  | **Colorectal Cancer** | | | | | | | **Colon Cancer** | | | | | | **Rectal Cancer** | | | | | | |
| --- | --- | --- | --- | --- | --- | --- | --- | --- | --- | --- | --- | --- | --- | --- | --- | --- | --- | --- | --- | --- |
|  | **15-34 Years** | **35-39 Years** | **40-49 Years** | **50-54 Years** | **55-69 Years** | **70+ Years** | **15-34 Years** | | **35-39 Years** | **40-49 Years** | **50-54 Years** | **55-69 Years** | **70+ Years** | | **15-34 Years** | **35-39 Years** | **40-49 Years** | **50-54 Years** | **55-69 Years** | **70+ Years** |
| **Females** |  |  |  |  |  |  |  | |  |  |  |  |  | |  |  |  |  |  |  |
| **Total Number of Incident Cases  (2003-2017) by Sex & Age** | 558 | 744 | 4,866 | 5,605 | 33,017 | 74,738 | 332 | | 441 | 2,743 | 3,241 | 21,029 | 53,636 | | 226 | 303 | 2,123 | 2,364 | 11,988 | 21,102 |
| **Mean Annual Number of Incident Cases by Sex & Age** | 37.2 | 49.6 | 324.4 | 373.7 | 2,201.1 | 4,982.5 | 22.1 | | 29.4 | 182.9 | 216.1 | 1,401.9 | 3,575.7 | | 15.1 | 20.2 | 141.5 | 157.6 | 799.2 | 1,406.8 |
| **Mean Annual Number of Incident Cases by Sex, Age & Period of Diagnosis** | | | | | | | | | | | | | | | | | | | | |
| 2003-2005 | 28.3 | 60.3 | 311.3 | 331.7 | 2,561.3 | 4,833.7 | 18.0 | | 39.0 | 176.7 | 200.3 | 1,622.0 | 3,418.3 | | 10.3 | 21.3 | 134.7 | 131.3 | 939.3 | 1,415.3 |
| 2006-2008 | 31.3 | 46.3 | 340.3 | 369.7 | 2,419.0 | 5,165.7 | 20.3 | | 28.3 | 194.3 | 207.7 | 1,533.3 | 3,641.3 | | 11.0 | 18.0 | 146.0 | 162.0 | 885.7 | 1,524.3 |
| 2009-2011 | 40.0 | 44.3 | 341.3 | 376.7 | 2,058.7 | 5,193.0 | 21.3 | | 27.0 | 191.7 | 217.7 | 1,318.7 | 3,736.3 | | 18.7 | 17.3 | 149.7 | 159.0 | 740.0 | 1,456.7 |
| 2012-2014 | 45.7 | 53.0 | 334.0 | 381.7 | 1,968.0 | 4,998.7 | 28.3 | | 29.3 | 192.0 | 215.3 | 1,242.7 | 3,599.7 | | 17.3 | 23.7 | 142.0 | 166.3 | 725.3 | 1,399.0 |
| 2015-2017 | 40.7 | 44.0 | 295.0 | 408.7 | 1,998.7 | 4,721.7 | 22.7 | | 23.3 | 159.7 | 239.3 | 1,293.0 | 3,483.0 | | 18.0 | 20.7 | 135.3 | 169.3 | 705.7 | 1,238.7 |
| **Mean Annual Age-Specific Incidence Rate by Sex & Age** | 1.1 | 5.0 | 14.2 | 34.7 | 82.2 | 200.8 | 0.7 | | 2.9 | 8.0 | 20.0 | 52.4 | 144.1 | | 0.4 | 2.1 | 6.2 | 14.6 | 29.8 | 56.7 |
| **Mean Annual Age-Specific Incidence Rate by Sex, Age & Period of Diagnosis** | | | | | | | | | | | | | | | | | | | | |
| 2003-2005 | 0.8 | 4.9 | 13.9 | 35.1 | 96.5 | 216.0 | 0.5 | | 3.2 | 7.9 | 21.2 | 61.1 | 152.8 | | 0.3 | 1.7 | 6.0 | 13.9 | 35.4 | 63.3 |
| 2006-2008 | 0.9 | 4.2 | 14.2 | 37.6 | 91.1 | 218.9 | 0.6 | | 2.5 | 8.1 | 21.1 | 57.7 | 154.3 | | 0.3 | 1.6 | 6.1 | 16.5 | 33.3 | 64.6 |
| 2009-2011 | 1.2 | 4.8 | 14.0 | 35.4 | 79.3 | 205.3 | 0.6 | | 2.9 | 7.9 | 20.4 | 50.8 | 147.7 | | 0.6 | 1.9 | 6.1 | 14.9 | 28.5 | 57.6 |
| 2012-2014 | 1.4 | 6.2 | 14.6 | 32.5 | 74.8 | 189.7 | 0.8 | | 3.4 | 8.4 | 18.4 | 47.2 | 136.6 | | 0.5 | 2.8 | 6.2 | 14.2 | 27.6 | 53.1 |
| 2015-2017 | 1.2 | 4.9 | 14.2 | 32.7 | 70.1 | 178.5 | 0.7 | | 2.6 | 7.7 | 19.2 | 45.4 | 131.7 | | 0.5 | 2.3 | 6.5 | 13.6 | 24.8 | 46.8 |
| **Males** |  |  |  |  |  |  |  | |  |  |  |  |  | |  |  |  |  |  |  |
| **Total Number of Incident Cases  (2003-2017) by Sex & Age** | 638 | 819 | 6,044 | 7,841 | 55,852 | 80,289 | 372 | | 424 | 3,085 | 3,846 | 30,622 | 52,659 | | 266 | 395 | 2,959 | 3,995 | 25,230 | 27,630 |
| **Mean Annual Number of Incident Cases by Sex & Age** | 42.5 | 54.6 | 402.9 | 522.7 | 3,723.5 | 5,352.6 | 24.8 | | 28.3 | 205.7 | 256.4 | 2,041.5 | 3,510.6 | | 17.7 | 26.3 | 197.3 | 266.3 | 1,682.0 | 1,842.0 |
| **Mean Annual Number of Incident Cases by Sex, Age & Period of Diagnosis** | | | | | | | | | | | | | | | | | | | | |
| 2003-2005 | 31.3 | 60.0 | 361.7 | 470.7 | 4,178.7 | 4,523.3 | 19.7 | | 34.3 | 183.3 | 226.3 | 2,321.3 | 2,954.3 | | 11.7 | 25.7 | 178.3 | 244.3 | 1,857.3 | 1,569.0 |
| 2006-2008 | 36.3 | 61.7 | 426.3 | 505.7 | 4,139.3 | 5,347.7 | 22.3 | | 30.7 | 223.7 | 252.3 | 2,247.3 | 3,492.3 | | 14.0 | 31.0 | 202.7 | 253.3 | 1,892.0 | 1,855.3 |
| 2009-2011 | 40.7 | 53.3 | 439.3 | 511.0 | 3,619.3 | 5,650.7 | 24.0 | | 25.3 | 212.3 | 251.3 | 1,994.0 | 3,674.0 | | 16.7 | 28.0 | 227.0 | 259.7 | 1,625.3 | 1,976.7 |
| 2012-2014 | 44.7 | 48.3 | 404.3 | 539.0 | 3,326.3 | 5,787.7 | 24.3 | | 27.7 | 203.0 | 256.7 | 1,806.7 | 3,811.7 | | 20.3 | 20.7 | 201.3 | 282.3 | 1,519.7 | 1,976.0 |
| 2015-2017 | 59.7 | 49.7 | 383.0 | 587.3 | 3,353.7 | 5,453.7 | 33.7 | | 23.3 | 206.0 | 295.3 | 1,838.0 | 3,620.7 | | 26.0 | 26.3 | 177.0 | 292.0 | 1,515.7 | 1,833.0 |
| **Mean Annual Age-Specific Incidence Rate by Sex & Age** | 1.2 | 5.4 | 11.7 | 48.1 | 144.4 | 319.1 | 0.5 | | 2.8 | 8.8 | 23.6 | 79.2 | 209.3 | | 0.5 | 2.6 | 8.4 | 24.5 | 65.2 | 109.8 |
| **Mean Annual Age-Specific Incidence Rate by Sex, Age & Period of Diagnosis** | | | | | | | | | | | | | | | | | | | | |
| 2003-2005 | 0.9 | 4.6 | 11.0 | 49.7 | 96.5 | 216.0 | 0.4 | | 2.6 | 7.9 | 23.9 | 90.7 | 222.7 | | 0.3 | 2.0 | 7.7 | 25.8 | 72.6 | 118.3 |
| 2006-2008 | 1.0 | 5.3 | 12.2 | 51.1 | 91.1 | 218.9 | 0.4 | | 2.7 | 9.0 | 25.5 | 87.6 | 230.6 | | 0.4 | 2.7 | 8.1 | 25.6 | 73.8 | 122.5 |
| 2009-2011 | 1.2 | 5.7 | 12.2 | 47.5 | 79.3 | 205.3 | 0.5 | | 2.7 | 8.5 | 23.4 | 79.8 | 212.0 | | 0.5 | 3.0 | 9.0 | 24.2 | 65.0 | 114.1 |
| 2012-2014 | 1.3 | 5.7 | 11.5 | 45.5 | 74.8 | 189.7 | 0.5 | | 3.2 | 8.7 | 21.6 | 71.5 | 202.9 | | 0.6 | 2.4 | 8.6 | 23.9 | 60.1 | 105.2 |
| 2015-2017 | 1.6 | 5.5 | 11.4 | 46.4 | 70.1 | 178.5 | 0.7 | | 2.6 | 9.8 | 23.3 | 67.0 | 187.2 | | 0.7 | 2.9 | 8.4 | 23.1 | 55.2 | 94.8 |

***Legend Supplemental Table 1:*** *Age-specific incidence rates = Cases per 100,000*

***Supplemental Table 2****:* *Number of Incident Cases and Age-Standardized Incidence Rates of Colorectal, Colon and Rectal Cancer Stratified by Sex and by Sex & Period of Diagnosis*

|  | **Females** |  |  | **Males** |  |  |
| --- | --- | --- | --- | --- | --- | --- |
|  | **Colorectal Cancer** | **Colon Cancer** | **Rectal Cancer** | **Colorectal Cancer** | **Colon Cancer** | **Rectal Cancer** |
| **Total Number of Incident Cases (2003-2017) by Sex** | 119,528 | 81,422 | 38,106 | 151,483 | 91,008 | 60,475 |
| **Mean Annual Number of Incident Cases by Sex** | 7,969 | 5,428 | 2,540 | 10,099 | 6,067 | 4,032 |
| **Mean Annual Number of Incident Cases by Sex & Period of Diagnosis** | | | | | | |
| 2003-2005 | 8,127 | 5,474 | 2,652 | 9,626 | 5,739 | 3,886 |
| 2006-2008 | 8,372 | 5,625 | 2,747 | 10,517 | 6,269 | 4,248 |
| 2009-2011 | 8,054 | 5,513 | 2,541 | 10,314 | 6,181 | 4,133 |
| 2012-2014 | 7,781 | 5,307 | 2,474 | 10,150 | 6,130 | 4,020 |
| 2015-2017 | 7,509 | 5,221 | 2,288 | 9,887 | 6,017 | 3,870 |
| **Mean Annual Incidence Rate by Sex (ASR** **Europe 1976) by Sex** | 30.3 | 19.9 | 10.3 | 49.6 | 29.2 | 20.3 |
| **Mean Annual Incidence Rate by Sex & Period of Diagnosis** (**ASR** **Europe 1976**) | | | | | | |
| 2003-2005 | 33.0 | 21.6 | 11.3 | 53.0 | 31.3 | 21.7 |
| 2006-2008 | 32.7 | 21.3 | 11.4 | 54.2 | 31.9 | 22.3 |
| 2009-2011 | 30.3 | 20.0 | 10.2 | 50.4 | 29.6 | 20.8 |
| 2012-2014 | 28.9 | 19.0 | 9.9 | 47.4 | 28.0 | 19.4 |
| 2015-2017 | 27.0 | 18.0 | 8.9 | 44.0 | 26.1 | 18.0 |
| **Mean Annual Incidence Rate by Sex (ASR** **World) by Sex** | 20.3 | 13.2 | 7.1 | 33.2 | 19.2 | 13.9 |
| **Mean Annual Incidence Rate by Sex & Period of Diagnosis** (**ASR** **World**) | | | | | | |
| 2003-2005 | 22.1 | 14.4 | 7.8 | 35.5 | 20.6 | 14.9 |
| 2006-2008 | 21.9 | 14.1 | 7.8 | 36.2 | 20.9 | 15.3 |
| 2009-2011 | 20.2 | 13.2 | 7.0 | 33.6 | 19.4 | 14.2 |
| 2012-2014 | 19.4 | 12.6 | 6.8 | 31.6 | 18.3 | 13.3 |
| 2015-2017 | 18.1 | 11.9 | 6.2 | 29.6 | 17.2 | 12.4 |

***Legend Supplemental Table 2:*** *ASR = Age-standardized rate;
Age-standardized and age-specific incidence rates = Cases per 100,000*
