# Supplementary material for: Temporal trends in age- and stage-specific incidence of colorectal adenocarcinomas in Germany
Source: BMC Cancer. 2023 Dec 1;23:1180. doi: 10.1186/s12885-023-11660-1 (PMC10693075; doi:10.1186/s12885-023-11660-1)
Supplement: Supplementary file 4 — Additional file 4. Supplemental File 2: Joinpoint Regression Model Specifications. [file 12885_2023_11660_MOESM4_ESM.docx]

***Supplemental File 2:* Joinpoint Regression Model Specifications**

The trend analyses were carried out with joinpoint regression models (SEER Joinpoint Software 4.9.1.0). The independent variable was selected as “Year” with interval type “Annual”. The “age-specific rate / crude rate” was chosen as the dependent variable. Where applicable the “By Variable” was “Stage”.

The model selection was conducted via weighted Bayesian information criterion (BIC).

The heteroskedastic error option was set to “constant variance (homoskedasticity)”. Log transformation was set to default “Yes {ln(y) = xb}”. A maximum of three joinpoints was allowed. The default minimum number of observations from a joinpoint to either end and default minimum number of observations between two joinpoints was 2. The default number of points to place between adjacent observed x-values in the grid search was 0. APC confidence interval was set to the default parametric method. The advanced analysis tool was set to „no advanced analyses“.
